# Multimodal brain tumor image segmentation based on DenseNet

**DOI:** 10.1371/journal.pone.0286125

**Published:** 2024-01-18

**Authors:** Xiaoqin Wu, Xiaoli Yang, Zhenwei Li, Lipei Liu, Yuxin Xia

**Affiliations:** School of Medical Technology and Engineering, Henan University of Science and Technology, Luoyang, P. R. China; Guru Ghasidas Vishwavidyalaya: Guru Ghasidas University, INDIA

## Abstract

A brain tumor magnetic resonance image processing algorithm can help doctors to diagnose and treat the patient’s condition, which has important application significance in clinical medicine. This paper proposes a network model based on the combination of U-net and DenseNet to solve the problems of class imbalance in multi-modal brain tumor image segmentation and the loss of effective information features caused by the integration of features in the traditional U-net network. The standard convolution blocks of the coding path and decoding path on the original network are improved to dense blocks, which enhances the transmission of features. The mixed loss function composed of the Binary Cross Entropy Loss function and the Tversky coefficient is used to replace the original single cross-entropy loss, which restrains the influence of irrelevant features on segmentation accuracy. Compared with U-Net, U-Net++, and PA-Net the algorithm in this paper has significantly improved the segmentation accuracy, reaching 0.846, 0.861, and 0.782 respectively in the Dice coefficient index of WT, TC, and ET. The PPV coefficient index has reached 0.849, 0.883, and 0.786 respectively. Compared with the traditional U-net network, the Dice coefficient index of the proposed algorithm exceeds 0.8%, 4.0%, and 1.4%, respectively, and the PPV coefficient index in the tumor core area and tumor enhancement area increases by 3% and 1.2% respectively. The proposed algorithm has the best performance in tumor core area segmentation, and its Sensitivity index has reached 0.924, which has good research significance and application value.

## 1. Introduction

A brain tumor is a kind of cancerous disease occurring in the skull cavity, which can be generally divided into primary brain tumors and brain metastasis. Among them, glioma is one of the most common malignant tumors in brain tumors [1]. It is a tumor derived from glial cells, accounting for 40% ~ 45% of intracranial tumors. It is divided into two types: high-grade glioma (HGG) and low-grade glioma (LGG). Magnetic resonance (MR) imaging [2], as a non-invasive brain tumor imaging technology, can produce high-resolution and non-invasive brain images. It is one of the most commonly used tools for brain examination in clinical medicine. MRI consists of four modes, namely T1-weighted (T1), T2-weighted (T2), Flair, and T1-weighted contrast enhancement (T1ce) [3]. In clinical practice, doctors will combine the four modes of brain tumor images and their segmentation results to develop appropriate treatment plans. Due to the various shapes, sizes, and positions of brain gliomas, it takes a long time to manually mark the lesions only by doctors, and the previous experience of the doctors is highly required, which affects the follow-up treatment of patients. Therefore, how to segment brain tumor images accurately and quickly is the challenge and goal of all brain tumor segmentation researchers.

In recent years, with the development of machine learning, image-processing methods based on deep learning have attracted wide attention. Dvorák and Menze [4] proposed a local structure prediction method to improve the local segmentation ability of magnetic resonance images. Kamnitsas et al. [5] designed an efficient intensive training scheme by using 3D fully connected conditional random fields to effectively remove false positives and achieve better segmentation results. Xie Mingchao et al. [6] proposed a segmentation method composed of two series stages based on convolutional neural network feature extraction, which extracts complete features and uses classifiers for classification. Experiments show that this method can adapt to the differences in brain tumors and help improve segmentation accuracy. Zikic et al. [7] designed a 2D brain tumor segmentation network using the AlexNet network, which uses multi-channel intensity information from facets around each point to be labeled as the input of the network, which has the advantage of fast training speed, but no post-processing is applied to the output of CNN, which is not conducive to tumor boundary segmentation. Isensee et al. [8] proposed NNU-Net based on the traditional U-Net model by improving the data preprocessing process, which further improved the accuracy of brain tumor segmentation. He et al. [9] proposed a ResNet network model. By establishing the connection between the front layer and the back layer, the backpropagation of the gradient is enhanced, so that a deeper CNN network can be trained and a higher segmentation accuracy can be obtained. Ibtehaz and Rahman [10] combined a deep residual network (ResNet) and U-net model to deepen the depth of the model and improve the accuracy of segmentation, but at the same time caused obvious gradient disappearance. Different from the RESNET network model, DenseNet is a dense connection between all the front layers and the rear layers. Each coding dense block is connected to the corresponding decoding dense block through a jump connection, which promotes the gradient flow in the network and the transmission of spatial information required for decoding coding input, promotes the backpropagation of the gradient, makes the network easier to train, and reduces the number of parameters to a certain extent.

**Fig 1:**
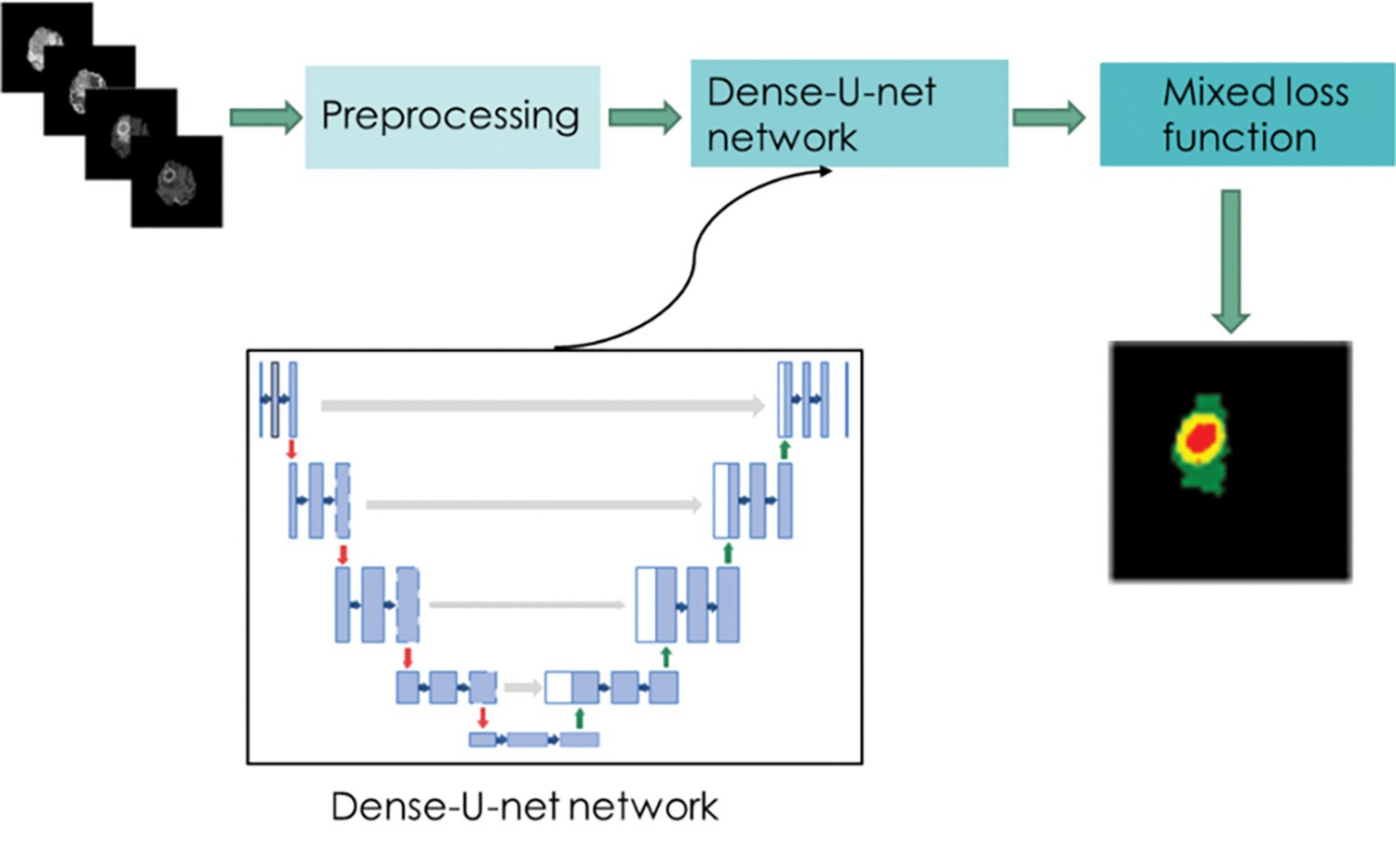
Algorithm framework of this paper.

This paper proposes an improved U-net [11] network segmentation algorithm, which combines the U-net network with the DenseNet network to obtain the DenseUnet network. The algorithm framework is shown in Fig 1. Dense blocks [12] are used to replace the convolution blocks in the traditional U-net network to enhance feature transmission and reduce overfitting. The robustness of the segmentation model is improved. This paper solves the problem of data imbalance caused by the large proportion of background feature pixels by clipping the background of the dataset. At the same time, a mixed loss function composed of the Binary Cross Entropy Loss function and Tversky coefficient is used to replace the original single cross-entropy loss function, which suppresses the influence of irrelevant features on the segmentation accuracy and can realize the accurate segmentation of brain tumor images. Experimental results show that the performance of the algorithm in this paper is better than U-net, U-Net++, PA-Net, and other morphs.

## 2. Method

### 2.1 Dataset

**Fig 2:**
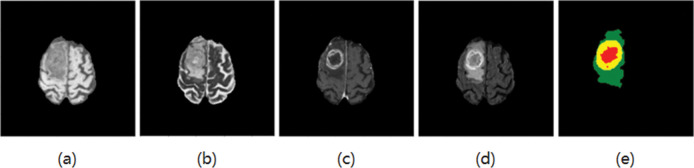
BraTS brain tumor datasets (a) T1, (b) T2, (c) T1ce, (d) Flair, and (e) GT.

The experimental data were obtained from the glioma public datasets BraTS2018 [13] and BraTS2019 [14] provided by the International Society for Medical Image Computing and Computer-Assisted Intervention (MICCAI). The training set in BraTs2019 has 285 cases (210 HGG patients, 75 LGG patients). There are 155 pictures in one MR sequence, and the size of each picture is 240x240. Each case has four modes (T1, T2, T1ce, FLAIR) and the corresponding standard segmentation label graph (GT), as shown in Fig 2. Compared with BraTs2018, the training set of BraTs2019 increased 49 cases of HGG and 1 case of LGG based on BraTs2018. The paper took the extra cases as the test set. There are 4 categories of labels in the datasets, label 0: healthy area; label 1: necrotic and non-enhanced tumor area; label 2: peritumoral edema area; label 4: enhanced tumor area. Brain tumors need to be divided into three parts: whole tumor area (WT), tumor enhancement area (ET), and tumor core area (TC). WT includes labels 1, 2, and 4; ET includes only labels 4; TC includes labels 1 and 4. An example of an MRI brain tumor image, as shown in Fig 2, where each color represents a label: the red area represents the necrotic and non-enhancing tumor range, the green area represents the peritumor edema range, and the yellow area represents the enhanced tumor range.

### 2.2 Image preprocessing

Since the four sequences of MRI are images of different modes with different signal-to-noise ratios and contrast, the data of each mode is normalized to zero mean [15] and unit standard deviation by adopting the Z-score method [16]. GT is the data we use to compare the effectiveness of the results, it is not standardized. As can be seen from Fig 2, the brain area is gray, while the background is black. The feature information of the background accounts for a large proportion of the whole image, while the proportion of the tumor area is small. When we train the model and classify each pixel, it will lead to data imbalance and affect the training effect. Therefore, to improve the segmentation effect, the black part was cut [17], that is, the original 240 × 240 MRI images were cropped to a size of 160 × 160. Since the four sequences in the datasets are three-dimensional images and the model is two-dimensional, the images and their corresponding GT are sliced into two-dimensional data during preprocessing. At the same time, to avoid the problem of class imbalance caused by excessive focus-free images in the datasets during model training, the focus-free images were discarded, and the slices of each mode were finally fused into multi-channels.

### 2.3 Model

**Fig 3:**
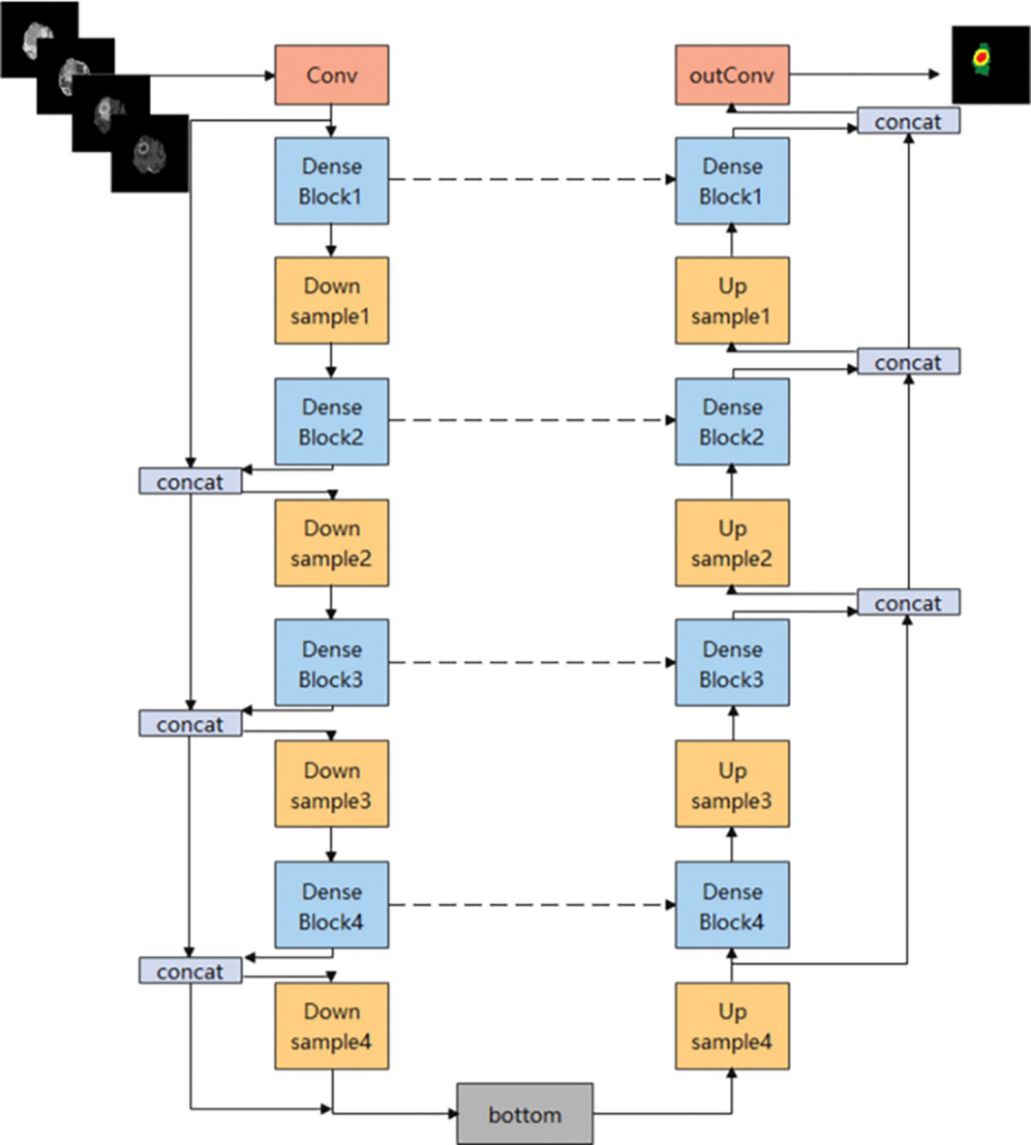
Dense-U-net network structure.

This paper designs a segmentation model based on the combination of U-Net and DenseNet based on retaining the U-Net network framework, dense blocks are added to the training network to strengthen the transmission of features, which can make more effective use of features and reduce overfitting. The network structure is shown in Fig 3.

DenseNet consists of DenseBlock and Transition Layers.

#### 2.3.1 DenseBlock

**Fig 4:**
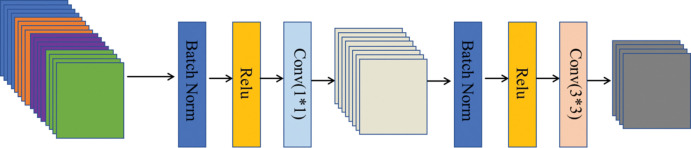
Dense block structure.

DenseNet achieves feature reuse through the connection of features on the channel. Each layer is connected with all previous layers in the channel dimension and together serves as the input of the network of the next layer, enhancing the transfer of features, as shown in Fig 4. The formula is expressed as:

$$x_{L}=H_{L}([x_{0},x_{1},x_{2},\ldots ,x_{L-1}])$$ (1)

The x_L_ is the input of the L layer; The x_0_, x_1_, x_2_,…,x_L−1_ represents the combination of the output of 0, 1, …, L-1 layer; H_L_ is defined as a composite function consisting of three modules: Batch Standardization [18] (BN), Rectified linear unit [19] (Relu), and 3×3 Convolution layer (Conv).

#### 2.3.2 Transition layers

Assuming that the network layer of DenseNet is l, the network contains $\frac{L(L+1)}{2}$ connections. According to the core idea of DenseNet, let the output dimension of layer L be y_L_, then the output dimension formula is expressed as follows:

$$y_{L}=y_{1}+y_{2}+\cdots +y_{L-1}$$ (2)

**Fig 5:**
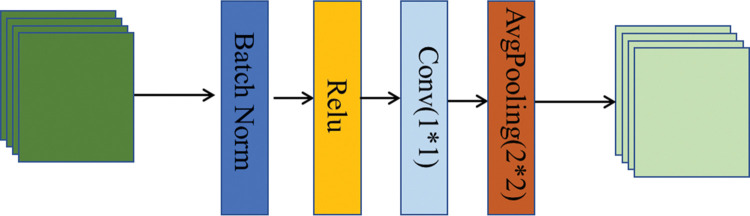
Structure diagram of a transition layer.

The main function of the transition layer is to connect two adjacent dense blocks, control the number of channels, and reduce the size of the feature map. The main structure is BN+Relu+1×1 of Conv+2×2 average pooling (AvgPooling), as shown in Fig 5. After the action of the compression model through the transition layer, the output dimension of each layer is m, and the final DenseBlock output dimension is αm, where α is the compression factor.

#### 2.3.3 Loss function

To solve the proportion imbalance of image tumor region and non-tumor region during model training, this study abandoned the single loss function and chose the mixed loss function composed of the Binary Cross Entropy Loss function (BCELoss) [20] and the Tversky coefficient [21]. The Binary Cross Entropy Loss function is defined as follows:

$${Loss}_{BCE}=-\frac{1}{N}\sum_{i=1}^{N}w*{\{(T}_{n}log(P_{n}))+(1-T_{n})*log(1-P_{n})\}$$ (3)

Where N is the number of samples; W is the weight, generally set to 1; T_n_ is the label value and P_n_ is the predicted value. The Tversky Loss formula is as follows:

$${Loss}_{Tver}=\frac{\left|A⋂B\right|}{\left|A⋂B|+\alpha |A-B|+\beta |B-A\right|}$$ (4)

Where A is the predicted label, B is the real label; |A−B| represents A false positives, |B−A| represents false negative, α and β weight to control false negatives and false positives respectively. In this paper, set α to 0.3 and β to 0.7. Finally, the mixed loss function is defined as follows:

$$Loss={Loss}_{BCE}+{Loss}_{Tver}$$ (5)

### 2.4 Evaluation indicators

This paper uses Dice similarity coefficient (Dice), precision (PPV), sensitivity, and Hausdorff_95 (95% HD) as the performance evaluation index, the specific calculation formula is as follows:

$$Dice=\frac{2TP}{FP+2TP+FN}$$ (6)

$$PPV=\frac{TP}{TP+FP}$$ (7)

$$Sensitivity=\frac{TP}{TP+FN}$$ (8)

$$Hausdorff=max[d_{xy},d_{yx}]=max\{{max}_{x\in X}{min}_{y\in Y}\|x-y\|,{max}_{x\in X}{min}_{y\in Y}\|y-x\|\}$$ (9)

Here, TP represents the number of positive classes correctly detected, FN represents the number of positive classes mistaken for negative classes, and FP represents the number of negative classes mistaken for positive classes. The range of the Dice coefficient value is 0 ~ 1, and the segmentation result is 1 as the best and 0 as the worst. PPV represents the proportion of samples with correct positive predictions. Sensitivity refers to the proportion of correctly predicted samples in the total positive samples. Hausdorff_95 is the last value multiplied by 95%, which represents the maximum value of the shortest distance between the segmentation result and the annotation result, and measures the maximum mismatch between the two points.

## 3. Results

### 3.1 Experimental results

**Fig 6:**
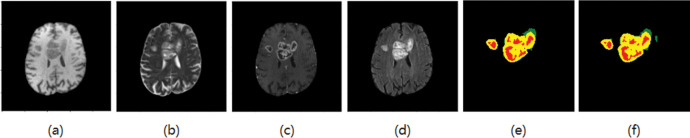
Segmentation results of the first group (a) T1 (b) T2 (c) T1ce (d) Flair (e) GT (f) The resulting diagram of the algorithm in this paper.

**Fig 7:**
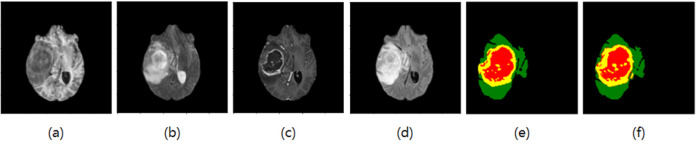
Segmentation results of the second group (a) T1 (b) T2 (c) T1ce (d) Flair (e) GT (f) The resulting diagram of the algorithm in this paper.

**Fig 8:**
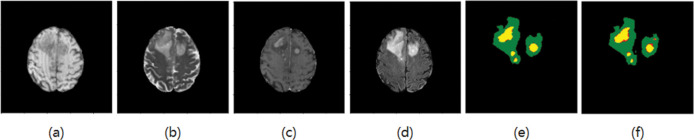
Segmentation results of the third group(a) T1 (b) T2 (c) T1ce (d) Flair (e) GT (f) The resulting diagram of the algorithm in this paper.

**Fig 9:**
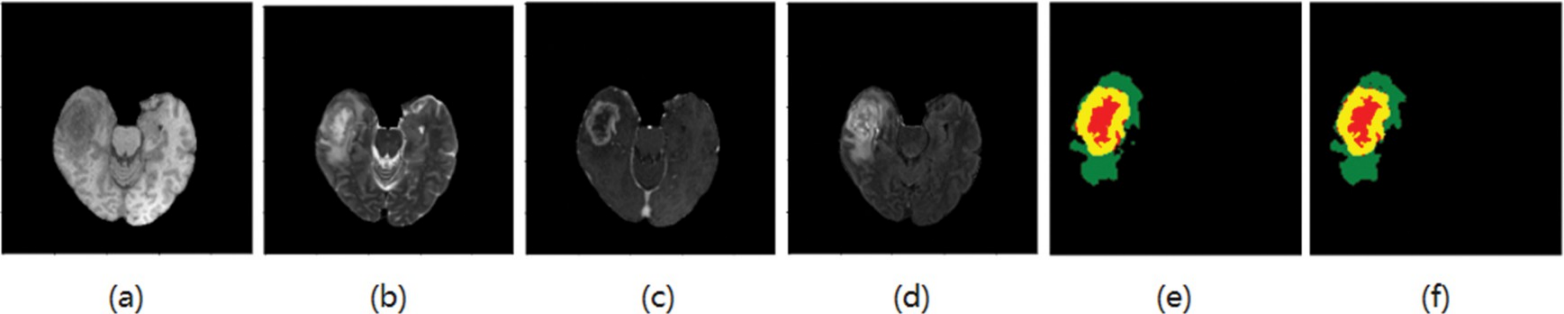
Segmentation results of the fourth group (a) T1 (b) T2 (c) T1ce (d) Flair (e) GT (f) The resulting diagram of the algorithm in this paper.

Adam optimizer is used in this paper, and weight decay is used as 1e-4. There are 55 epochs in total, the initial learning rate is 3e-4, the batch size is 32, and the activation function is Softmax. The segmentation results are shown in Figs 6–9, where T1, T2, T1ce, and Flair are the four modes of the original image, GT is the standard segmentation result with labels, and Pre is the resulting graph of the algorithm in this paper.

As shown in Figs 6 and 7, the edema area around the tumor can be accurately segmented, and the shape and contour segmentation of the enhanced tumor area is relatively complete, which proves the feasibility of the proposed algorithm. However, in the results of Figs 8 and 9, some small necrotizing and non-enhancing tumor areas were not segmented, which will be further improved in future work.

### 3.2 Analysis

**Fig 10:**
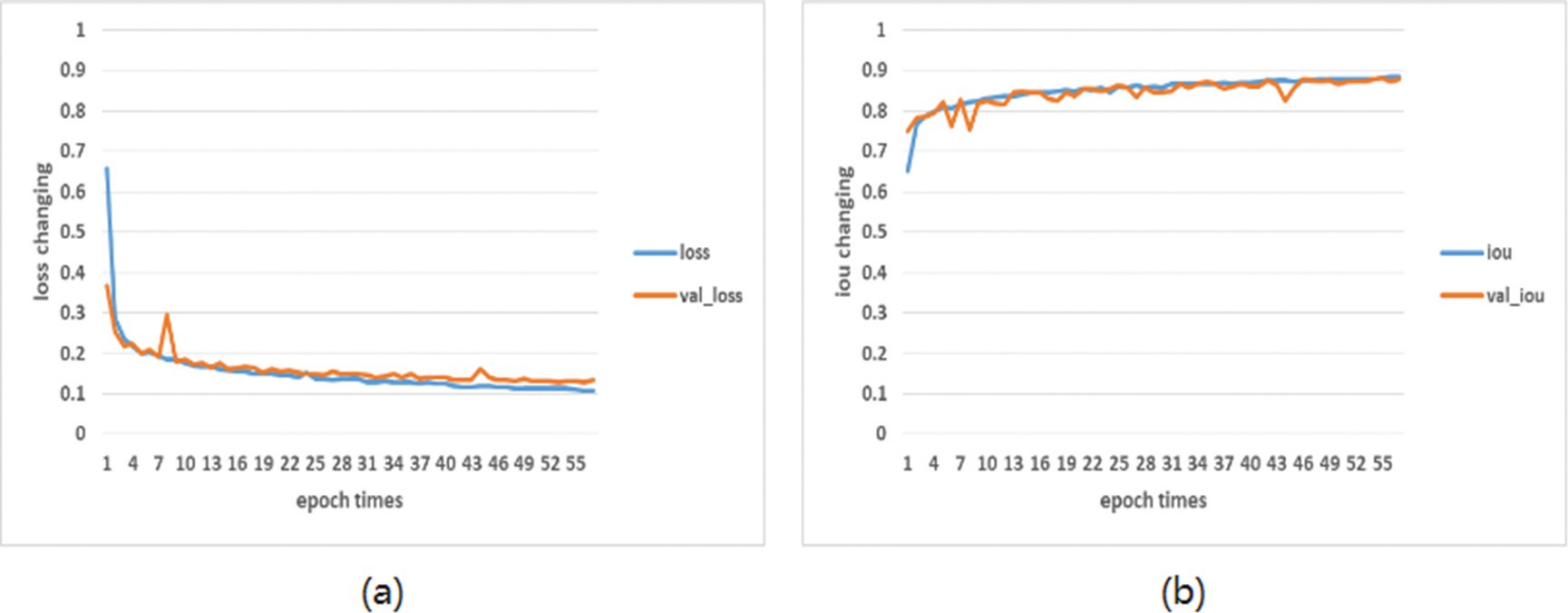
Curve changes of loss and iou of the training set and verification set (a) loss change curve (b) iou change curve.

The curve of the loss value and intersection over union [22] (iou, an evaluation index used to measure the accuracy of the target detector on a specific dataset) of the training set and the verification set with the epoch times during the training process of the improved model is shown in Fig 10(A) and 10(B). In figure (a), the abscissa represents the number of epochs, and the ordinate represents the value of loss changing with the epoch. In figure (b), the abscess represents the epoch times, and the ordinate represents the value of the iou changing with the epoch.

As shown in Table 1, compared with U-Net, U-Net++, and PA-Net the algorithm in this paper has significantly improved the segmentation accuracy, reaching 0.846, 0.861, and 0.782 respectively in the dice index of WT, TC, and ET, which exceeds 0.8%, 4.0%, and 1.4% compared with the traditional U-Net network. Excellent performance in tumor enhancement region segmentation; PPV index reached 0.849, 0.883, and 0.786 respectively, which was also greatly improved in TC segmentation of tumor enhancement region.

**Table 1:** Comparison of dice and PPV of segmentation effects of different models.

Model	Dice			PPV		
	WT	TC	ET	WT	TC	ET
U-Net	0.838	0.821	0.768	0.856	0.853	0.774
U-Net++	0.842	0.826	0.774	**0.866**	0.850	**0.791**
PA-Net	0.801	0.681	0.718	0.792	0.695	0.681
Our proposed	**0.846**	**0.861**	**0.782**	0.849	**0.883**	0.786

The sensitivity reached 0.887, 0.924, and 0.839 respectively, exceeding 1.9%, 0.7%, and 3.8% of the traditional U-Net network respectively; The Hausdorff distance is 2.611, 1.601, and 2.767, respectively, as shown in Table 2. The results show that the proposed algorithm has good segmentation performance.

**Table 2:** Comparison of sensitivity and Hausdorff of segmentation effects of different models.

Model	Sensitivity			Hausdorff		
	WT	TC	ET	WT	TC	ET
U-Net	0.868	0.917	0.801	2.627	1.804	2.814
U-Net++	0.868	0.912	0.827	2.638	1.722	2.814
PA-Net	0.80	0.675	0.759	4.611	4.136	4.923
Our proposed	**0.887**	**0.924**	**0.839**	**2.611**	**1.601**	**2.767**

The loss function proposed in this paper, which combines the Binary Cross Entropy Loss function and Tversky coefficient, improves the algorithm performance and segmentation accuracy compared with other mixed loss functions, as shown in Tables 3 and 4. It can be seen that the advantages of this paper compared with other mixed loss functions can effectively improve the accuracy of tumor segmentation. It can be seen that this paper is superior to other mixed loss functions and can effectively improve the accuracy of tumor segmentation.

**Table 3:** Comparison of Dice and PPV for segmentation effects of different loss functions.

Loss	Dice			PPV		
	WT	TC	ET	WT	TC	ET
*Loss*_*BCE*_+*Loss*_*jaccard*_	0.840	0.856	0.771	0.853	**0.889**	0.728
*Loss*_*BCE*_+*Loss*_*Dice*_	**0.847**	0.854	0.781	**0.873**	0.883	**0.804**
Our proposed	0.846	**0.861**	**0.782**	0.850	0.883	0.756

**Table 4:** Comparison of sensitivity and Hausdorff of segmentation effects of different loss functions.

Loss	Sensitivity			Hausdorff		
	WT	TC	ET	WT	TC	ET
*Loss*_*BCE*_+*Loss*_*jaccard*_	0.877	0.916	0.834	2.644	1.625	2.817
*Loss*_*BCE*_+*Loss*_*Dice*_	0.869	0.915	0.823	2.660	1.648	**2.755**
Our proposed	**0.887**	**0.924**	**0.839**	**2.611**	**1.607**	2.767

The Dice evaluation index of published literature was compared, as shown in Table 5, and the proposed method exceeded all the mentioned methods in TC segmentation and ET segmentation, reaching 0.86 and 0.78 respectively, showing a great improvement effect.

**Table 5:** Comparison of Dice evaluation indexes between different literature methods.

Methods	Dice		
	WT	TC	ET
Dong [19]	0.88	0.79	0.73
Rehman [20]	**0.90**	0.75	0.71
Havaei [3]	0.79	0.58	0.69
Pereira [21]	0.78	0.65	0.75
Chen [22]	0.85	0.70	0.61
LI [23]	0.87	0.77	0.72
Our proposed	0.84	**0.86**	0**.78**

## 4. Conclusions

In this study, a network model based on the combination of U-net and DenseNet is proposed to solve the problems of class imbalance and overfitting in multimodal brain tumor image segmentation. The convolution blocks in the U-net network are replaced by dense blocks, which can make more effective use of features and reduce overfitting. The mixed loss function consisting of the Binary Cross Entropy Loss function and the Tversky coefficient is used to suppress the impact of irrelevant features on segmentation accuracy. Experiments show that the segmentation accuracy of this algorithm has been improved compared with the U-net network model. In addition, the research in this paper is based on a 2D network, while the original images in the data set are 3D medical images. Due to the limitations of using 2D network to segment 3D image data, only 2D slices can be used for training, resulting in the model being unable to obtain the relationship information between layers, so future research will focus on 3D brain tumor image segmentation.

## Supporting information

S1 Appendix (DOCX)
