# Supplementary material for: Multimodal brain tumor image segmentation based on DenseNet
Source: PLoS One. 2024 Jan 18;19(1):e0286125. doi: 10.1371/journal.pone.0286125 (PMC10796062; doi:10.1371/journal.pone.0286125)
Supplement: S1 Appendix — (DOCX) [file pone.0286125.s001.docx]

**Appendix**

| Mathematical Symbols | Meaning |
| --- | --- |
| α | control false negative |
| β | control false positive |
